# Intense FDG Uptake around the Inguinal Surgical Mesh 5 Years after Operation: Case Report and Review of the Literature

**DOI:** 10.4274/Mirt.021917

**Published:** 2012-04-01

**Authors:** Tatiana Bahçeci, Gül Nihal Nursal, Mehmet Aydın

**Affiliations:** 1 Başkent University, Department of Nuclear Medicine, Ankara, Turkey

**Keywords:** Positron emission tomography, fluorodeoxyglucose F-18, surgical mesh, foreign-body reaction

## Abstract

We present the case of a 40-year-old man who underwent a FDG PET/CT study for restaging of renal cell carcinoma treated with left nephrectomy, for suspected metastasis in lung and retroperitoneal lymph nodes. The patient had a history of left inguinal hernia repair with implantation of mesh prosthesis 5 years ago. PET/CT image revealed linear intense FDG uptake in left inguinal region most likely corresponding to a persistent foreign body reaction. In this article, a case with an intense FDG uptake around mesh prosthesis after many years was reported, and a summary of the literature about surgical mesh and foreign body reaction causing FDG uptake was reviewed.

**Conflict of interest:**None declared.

## INTRODUCTION

Over the last few years, 18F-fluorodeoxyglucose (FDG) PET/CT has become increasingly widespread in the management of various types of cancer patients. Although FDG PET/CT has a high accuracy especially in certain clinical settings, FDG as a glucose analogue is a very sensitive but less specific tracer (1) Not only most tumor cells, but inflammatory cells have increased FDG uptake, which cause sometimes false positive results in cancer patients. One of these unexpected situations is surgical mesh, which causes FDG uptake corresponding to foreign body reaction or infection (2,3,4). In this article, a patient with intense FDG uptake around his left inguinal surgical mesh was reported and the review of the literature was presented.

## CASE REPORT

**Figure 1:**
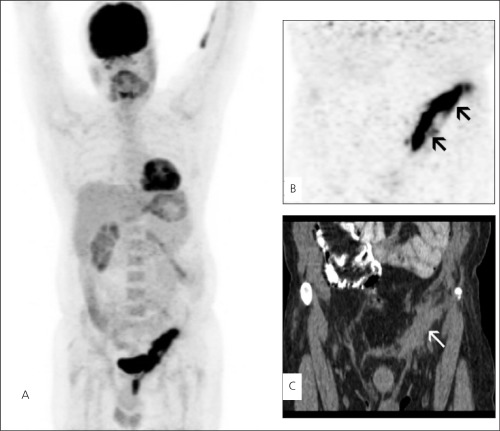
A, B, C. Maximum intensity projection (MIP) images (A) andcoronal PET image (B) show increased FDG uptake on left inguinalregion (arrows). Coronal CT image (C) demonstrates soft tissue thickening(white arrow)

**Figure 2:**
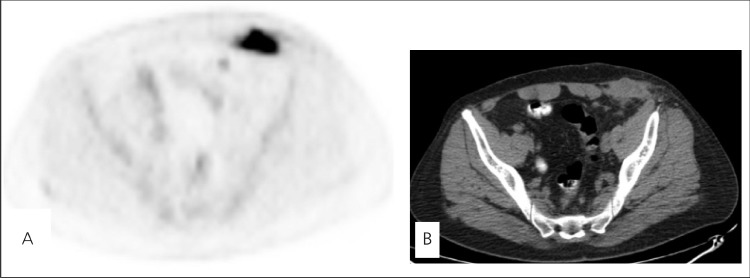
A, B. Axial PET image of the pelvic region shows an intenseFDG uptake at left anterior abdominal wall corresponding to repairedhernia region with surgical mesh implant, with a maximum standardizeduptake value (SUV) of 17.5. Axial CT image demonstrates softtissue thickening including a hypodense area

A 40-year-old man underwent a FDG PET/CT study for restaging of renal cell carcinoma for suspected metastasis in lung and retroperitoneal lymph nodes. He was treated with left nephrectomy two months ago. PET images were acquired 60 minutes after intravenous injection of 333 MBq (9 mCi) FDG nn PET/CT scanner (GE Discovery STE 8). PET images showed a slight FDG uptake over the left subcostal incision line due to recent left nephrectomy scar and an unexpected intense FDG uptake on left inguinal region (Figure 1a, b, c; Figure 2a, b). From the patient’s clinical history, we learned that he had a left inguinal hernia repair with implantation of mesh prosthesis 5 years ago. By now, the patient had no pain but a serous leakage from left inguinal region without any infection signs. This linear intense FDG uptake in left inguinal region suggested a persistent foreign body reaction. In this patient, PET/CT showed physiological FDG uptake on the rest of the body without any metastases.

## LITERATURE REVIEW AND DISCUSSION

FDG PET/CT is widely used for evaluation of various types of cancer patients. FDG is a glucose analogue and a non-specific agent for malignancy. Increased FDG uptake can be seen not only in malignant lesions, but also in benign inflammatory processes. Active granulomatous processes, infectious diseases and inflammatory reactions are well-known false positive causes for cancer patients (1).

Surgical mesh is a woven fabric used for chest wall reconstruction, strengthening tissues; provide support for internal organs, and to treat surgical or traumatic wounds. Surgical mesh is often used in hernia repair and is placed on or under the damaged area in the abdomen. As with any surgical implant, some complications can occur, including infection, inflammation and tissue damage (5).

FDG uptake in surgical mesh has been reported previously (2,3,4,6,7) In the literature, there are some cases that show FDG uptake around mesh prosthesis due to persistent foreign body reaction that occurred up to 10 and 25 years after implantation (2,3,4) It can be considered as a pitfall of PET/CT, such a source of false positive results should be carefully avoided by reviewing the patient’s history. In our case, an intense FDG uptake in the absence of infection strongly suggested persistent foreign body reaction. In a rat model study, immunohistochemical analysis revealed macrophage predominance cell type in the mesh prostheses-dependent chronic inflammatory infiltrate (6).

In literature, there are several cases that show FDG uptake corresponding to foreign body reaction. Such uptake has been reported in conjunction with mesh and teflon prostheses, breast silicone, catheter, arthroplasty, and the other foreign bodies (8,9,10,11,12,13,14,15,16,17,18,19,20,21,22,23,24,25). The FDG uptake mechanism is considered as a foreign body granulomatous reaction with inflammation and fibrosis. Careful correlation with the patient’s history and correlative imaging techniques such as CT are recommended to avoid misdiagnosing malignancy.In summary, this case report shows a very intense FDG uptake around mesh prostheses suggesting foreign body reaction, occurred 5 years after inguinal hernia repair. We have reviewed FDG uptake in mesh-induced foreign body reaction with this case report, to emphasize the importance of the clinical history during interpretation of PET/CT studies.
